# Effect of a Phytochemical-Rich Olive-Derived Extract on Anthropometric, Hematological, and Metabolic Parameters

**DOI:** 10.3390/nu16183068

**Published:** 2024-09-11

**Authors:** Anna Aiello, Luana Calabrone, Douglas M. Noonan, Paola Corradino, Sara Nofri, Simone Cristoni, Giulia Accardi, Giuseppina Candore, Calogero Caruso, Angelo Zinellu, Adriana Albini

**Affiliations:** 1 Laboratory of Immunopathology and Immunosenescence, Department of Biomedicine, Neuroscience and Advanced Diagnostics, University of Palermo, 90134 Palermo, Italy; anna.aiello@unipa.it (A.A.); giulia.accardi@unipa.it (G.A.); giuseppina.candore@unipa.it (G.C.); calogero.caruso@unipa.it (C.C.); 2 ISB—Ion Source & Biotecnologie Srl, Rho, 20017 Milan, Italy; luana.calabrone@gmail.com (L.C.); simone.cristoni@gmail.com (S.C.); 3 Unit of Molecular Pathology, Biochemistry and Immunology, IRCCS MultiMedica, 20138 Milan, Italy; douglas.noonan@gmail.com; 4 Department of Biotechnology and Life Sciences, University of Insubria, 21100 Varese, Italy; 5 European Institute of Oncology (IEO), Istituto di Ricovero e Cura a Carattere Scientifico (IRCCS), 20141 Milan, Italy; paola.corradino@ieo.it; 6 University of Florence, 50139 Florence, Italy; sara.nofri@googlemail.com; 7 Department of Biomedical Sciences, University of Sassari, 07100 Sassari, Italy; azinellu@uniss.it

**Keywords:** extra virgin olive oil, olive mill wastewater, phytochemicals, polyphenols, antioxidants, antiaging, metabolic syndrome, cancer

## Abstract

Background: Extra virgin olive oil is a fundamental component of the Mediterranean diet. It contains several molecules that sustain human well-being by modulating cellular metabolism and exerting antioxidant, anti-inflammatory, and anti-ageing effects to protect normal tissues, and it can exert anti-angiogenic and pro-apoptotic effects on cancer cells. Metabolites found in different parts of the olive tree, including leaves, also possess properties that might help in cancer prevention and promote wellness in aging. Olive mill wastewater (OMWW), a liquid residue produced during olive oil extraction, represents an environmental issue. However, it is rich in phytochemicals with potential beneficial properties. Dietary supplements based on OMWW can be produced for nutritional supplementation with advantages to the ecology. Purpose: This work aims to measure hematochemical, anthropometric, and metabolomic parameters in volunteers taking an OMWW dietary supplement, Oliphenolia^®^ (OMWW-OL). Methods: The supplementation of OMWW-OL 25 mL twice daily for 30 days was tested on a pilot cohort of volunteers with characteristics close to metabolic syndrome. Hematochemical, anthropometric, serum biomarkers and serum metabolomic parameters were analyzed before the intervention, at 30 days, and 30 days after stopping consumption. Results: A total of 29 volunteers were enrolled, and 23 completed the study. The participants’ parameters at baseline were measured, and then twice daily at 30 days of treatment and 30 days after assumption discontinuation. Although treatment was with an olive derivative, their weight did not increase. Their body mass index, instead of augmenting, slightly decreased, particularly in the women. Also, hydration increased, especially in the women, while blood pressure, glycemia, and insulin decreased. Cholesterol, high-density lipoproteins, and triglycerides were stable, and LDL levels decreased, while vitamin D levels, alongside calcium, perceptibly increased. Albumin also increased. All the values were in support of an equilibrium, with no damaging effects. By mass spectrometry analysis, we also found favorable changes in the vitamin D/histamine and homocysteine/methionine ratios, an increase in a new metabolite of unknown formula, and the vitamin D/unknown metabolite ratio. Conclusions: Supplementation of OMWW-OL has no detrimental effects and might imply the beneficial modulation of several biological parameters. Although this is a small pilot study, with limited potency, it preliminarily suggests that the OMWW extract use could be potentially valuable for people at risk of metabolic syndrome. Some of these parameters could also be relevant in supporting healthy ageing and in cancer prevention.

## 1. Introduction

An integral part of the Mediterranean diet, extra virgin olive oil (EVOO) is high in monounsaturated fatty acids, which are regarded as advantageous for protection against diseases [1]. Since EVOO seems to improve omega-3 polyunsaturated fatty acids’ oxidative stability, it might have positive effects on age-related conditions [2]. It is also a good source of antioxidants, including polyphenols and vitamin E, which may help protect against oxidative damage in the body, the cause of several chronic degenerative diseases [3,4]. EVOO reduces the risk of cardiovascular pathologies, type 2 diabetes, non-alcoholic fatty liver disease (NAFLD), neurodegenerative diseases, and some cancers by displaying anti-inflammatory and antioxidant effects [5,6,7,8,9,10,11]. Olive trees can produce useful metabolites in a variety of tissues, including the leaves. Olive mill wastewater (OMWW) is a large-scale liquid waste product released during olive oil extraction. It is an environmental concern on the one hand, but on the other, it is a rich source of phytochemicals that may have beneficial effects [6,12,13]. Hydroxytyrosol is the most common polyphenol in OMWW extracts aside from verbascoside and oleuropein, which are also abundant [14]. It has been shown that polyphenols control inflammation and oxidative stress [15], regulate gene expression in metabolic pathways, and have the potential to improve insulin sensitivity [16]. In this way, polyphenols could play an important role in the prevention of metabolic syndrome, which is characterized by hypertension, hyperglycemia, hyperinsulinemia, dyslipidemia, and obesity [17,18]. Thanks to pharmacometabolomics [19,20,21], the personalization of drug therapy is possible through the comprehensive analysis of metabolites in a patient’s biological fluids. Metabolomics can provide important information on the effect of clinical treatments on the metabolism, and therefore, mass spectrometry (MS) has taken a central role in personalized and precision medicine [22,23,24,25].

In this work, we report the application of a dietary strategy to study the effects of an OMWW-derived supplement, Oliphenolia^®^ (OMWW-OL), on different anthropometric and serum parameters at distinct time points. Our aim was to measure hematochemical, anti-inflammatory, antioxidant, and physiological parameters and to assess metabolic pathways before initiation (T0), after 30 days of nutritional intervention (T1), and 30 days after the end of the nutritional intervention (T2).

## 2. Materials and Methods

### 2.1. Study Design

**Figure 1:**
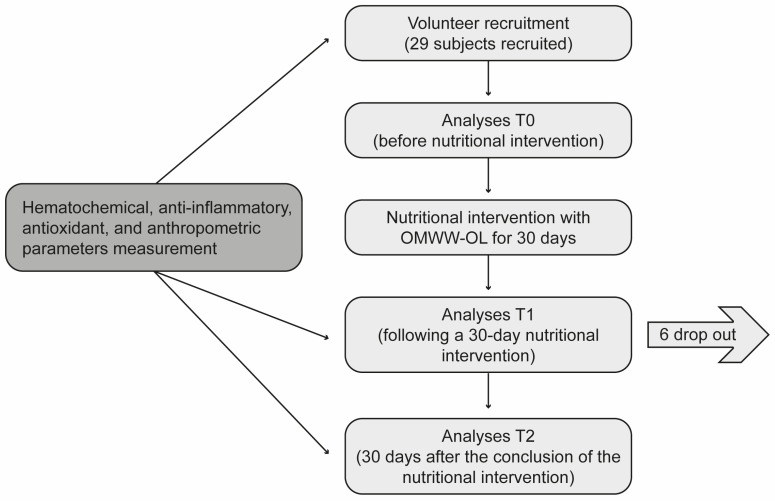
The flowchart of the study design. The study protocol was approved by the Ethics Committee. During a 7-day washout period, the participants were required to abstain from extra virgin olive oil and food supplements containing polyphenols. The nutritional intervention consisted of the consumption of the Oliphenolia^®^ dietary supplement preparation (25 mL) twice a day, 30 min before the main meals (lunch and dinner), for 30 days, with no other restriction from the usual diet.

This was a single-arm longitudinal interventional pilot study to evaluate the effect of OMWW-OL on a cohort of volunteers with characteristics close to metabolic syndrome as a pilot study. Participants were enrolled in Western Sicily within the project “Nutraceutical effects of olive products: Role in the achievement of longevity”. The dietary intervention consisted in the consumption of 25 mL of OMWW-OL twice a day, 30 min before the main meals (lunch and dinner) for 30 days. Before starting the nutritional intervention, a 7-day washout period was carried out, during which the participants were required to abstain from EVOO and food supplements containing polyphenols. The flowchart of the study design is reported in Figure 1.

### 2.2. Study Participants

The subjects enrolled had at least one of the following inclusion criteria: slight dyslipidemia (TC 190–240 mg/dL, triglyceride level ≥ 150 mg/dL); a waist circumference ≥ 102 cm in men and ≥88 cm in women; and altered glucose tolerance (fasting blood glucose ≥ 100 mg/dL). The exclusion criteria were as follows: any treatment for specific diseases, including drugs to treat metabolic disorders; the diagnosis of severe systemic disease; a history of treatment with statins or similar drugs or other liposoluble or hypoglycemic drugs; restrictive dietary requirements (e.g., gluten-free diet). The onset of gastrointestinal disorders during the intervention determined the prompt exclusion from the study. No restriction related to gender was considered.

### 2.3. Ethical Considerations

Before enrollment, each recruited subject was fully informed about the purposes and procedures of the experiment, and their informed consent was obtained. To respect privacy, patients’ names were anonymized by assigning each one an encoded alpha-numeric identification code in accordance with the General Data Protection Regulation (GDPR, EU Regulation 2016/679) concerning the protection of individuals with regard to the processing of personal data, as well as the free circulation of such data. The study protocol was conducted in accordance with the 1964 Declaration of Helsinki and its later amendments, and the institutional Ethics Committee (Policlinico Paolo Giaccone, University Hospital) approved the study protocol (Ruolo dei prodotti dell’olivo nella prevenzione delle malattie età correlate/Role of olive products in the prevention of age-related diseases) on 18 February 2019, and the reference number is 02/2019.

### 2.4. Data Collection

The demographic, clinical, and anamnestic data of the probands were collected by a group of well-trained biologists and physicians from the University of Palermo. Additionally, a weekly food diary was provided to record data on their dietary intake. A database in Excel was created to collect and handle all participants’ data and information. To obtain informative and reproducible analyses, anthropometric measures were obtained while subjects wore light clothes and no shoes. Body weight was measured using an electronic scale calibrated in kilograms. Height was measured with the subject in the supine position with a stadiometer. The bioelectrical impedance analysis (BIA) was also performed in the supine position, wearing light clothes and barefoot. The resistance and the reactance, both in ohms and the phase angle, were reported; the body compartment measurements (in percentage by weight and kg/m^2^) were estimated using regression equations by Bodygram^®^Plus 1.1.4.4 software (AKERN^®^, Pisa, Italy). The body mass index (BMI, kg/m^2^) was calculated using anthropometric measurements, such as height (m) and weight (kg).

### 2.5. Analysis of Hematochemical Parameters

Overnight fasting blood samples were collected in the morning. The blood was collected in specific tubes without additives in order to separate the serum samples, which were obtained after centrifugation at 2500 rpm for 15 min at 4 °C, rapidly frozen, and stored at −80 °C for further tests. All hematochemical analyses were measured by standard biochemical assays. The concentration of serum low-density lipoprotein (LDL) cholesterol was calculated using the Friedewald equation: LDL = total cholesterol-high-density lipoproteins (HDLs) − (triglycerides/5). Samples before initiation (T0), after 30 days of nutritional intervention (T1), and 30 days after the end of the nutritional intervention (T2) were also collected for spectrometry analysis.

### 2.6. Evaluation of the Antioxidative Effect

Serum samples from the patients were also used for the determination of human oxidized LDL (oxLDL) quantity, using a specific ELISA kit (Cusabio, Houston, TX, USA) following the manufacturer’s instructions. The competitive inhibition enzyme immunoassay method was used in this assay. The microtiter plate was pre-coated with oxLDL. Standards or samples were added to the appropriate microtiter plate wells with a horseradish peroxidase-conjugated antibody preparation specific for oxLDL. The pre-coated oxLDL and oxLDL in the samples were used to start a competitive inhibition reaction. The absorption was determined with a microplate spectrophotometer (Tecan, Switzerland) at 450 nm. The results were interpreted by constructing a dose/response curve according to the standards provided in the kit.

### 2.7. Liquid Chromatography SANIST Mass Spectrometry

Serum metabolites were measured with an untargeted liquid chromatography (LC)-MS SANIST platform. The preliminary MS biomarker identification was obtained based on the accuracy of the derived molecular mass data (*m*/*z*, mass error: 10 ppm), and the ratios of the relative abundance of the different metabolites (i.e., “metabolite ratio”) were calculated [26,27]. Thanks to the calculated ratios, metabolites were classified using an unsupervised method [26]. To 300 μL of serum, 600 μL of acetonitrile (CH_3_CN, Sigma Aldrich, Milan, Italy) was added to precipitate proteins and centrifuged at 13,000× *g* for 10 min at room temperature. An aliquot of supernatant (600 μL) was collected and spiked with 100 μL of Ultramark polymer solution (10 ng/mL) to check the stability of the LC-MS signal during analysis. A SpeedVac (ThermoFisher, San Jose, CA, USA) was used to dry the remaining 300 μL; 100 μL of doubly distilled water/methanol (1:1, *v*/*v*; Sigma Aldrich, Milan, Italy) (1:1, *v*/*v*) solution was used to resuspend each sample, and 10 µL was added to the internal standard reserpine (10 μL, final concentration 10 ng/mL, Sigma Aldrich, Milan, Italy). The samples were directly injected into the LC-MS apparatus. A reverse-phase C-18 LC column (50 × 2.1 mm; particle size, 5 µm; pore size, 100 Å; Phenomenex, [Torrance, CA OR San Jose, CA], USA) was employed. The mobile phases consisted of (A) water containing 0.2% *v*/*v* formic acid (HCOOH, Sigma Aldrich, Milan, Italy), and (B) CH_3_CN (100%), and the separation was achieved using an elution gradient. The flow rate was 0.25 mL/min, and the injection volume was 15 µL. Full-scan spectra were acquired in the mass range *m*/*z* 40–800. Tandem mass spectrometry (MS/MS) experiments on the serum samples were performed with collision-induced dissociation, using helium as the collision gas. The precursor ions were isolated and fragmented (isolation windows, ±0.3 *m*/*z*; collision energy, 30% of its maximum value, which was 5 V peak to peak), and then product ions were obtained and analyzed by an Orbitrap mass analyzer with a highly accurate *m*/*z* ratio (mass resolution *m*/*z* 15,000; mass error < 10 ppm). Compound identification was made based on fragmentation patterns and a mass search database (NIST14) library following the European Union database match mode (EU directive 2002/657/EC) [28]. The chromatographic data were elaborated using the Xcalibur Quan Browser (Version 4.1.31.9). The SANIST data elaboration tool was used to predict the metabolomic pathways altered in the serum samples of the participating subjects [29].

### 2.8. Statistical Analysis

Graphics and statistical analyses were performed using GraphPad Prism Software (GraphPad Software, Inc., San Diego, CA, USA, Ver. 10.0.2). The figures show the single values at T0, T1, and T2. The *Y*-axis reports the values of the analyzed biomarker. Statistics are performed in comparison between the average of T0/T1 ± SEM, T1/T2 ± SEM T0/T2 ± SEM. Significance was calculated from the mean ± SD and analyzed using Student’s *t*-test and one-way Anova. A value of *p* < 0.05 was considered statistically significant. * *p* < 0.05, ** *p* < 0.01, *** *p* < 0.001, and **** *p* < 0.0001.

## 3. Results

### 3.1. Anthropometric, Hematochemical, Inflammatory and Oxidative Parameters

**Figure 2:**
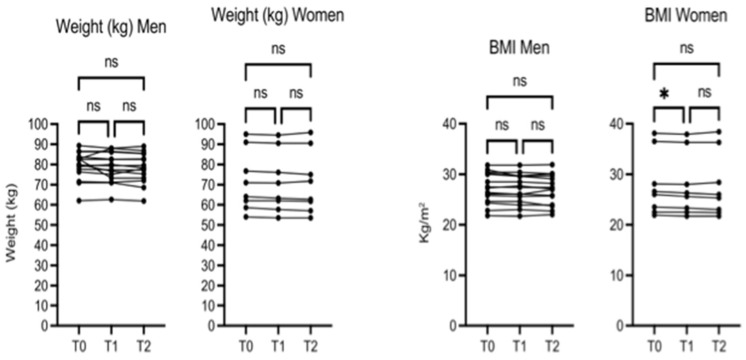
OMWW-OL consumption effects on weight and BMI in volunteers. The images show the single values at T0, T1, and T2. There were no differences in the men’s weight and BMI values; in the women, the BMI showed a slight decrease at 30 days (T1), returning to the value of T0 after 60 days (T2). The *Y*-axis reports the values of the analyzed biomarker. Statistics are performed in comparison between the average of T0/T1 ± SEM, T1/T2 ± SEM, T0/T2 ± SEM. * *p* < 0.05, ns: non-significant.

**Figure 3:**
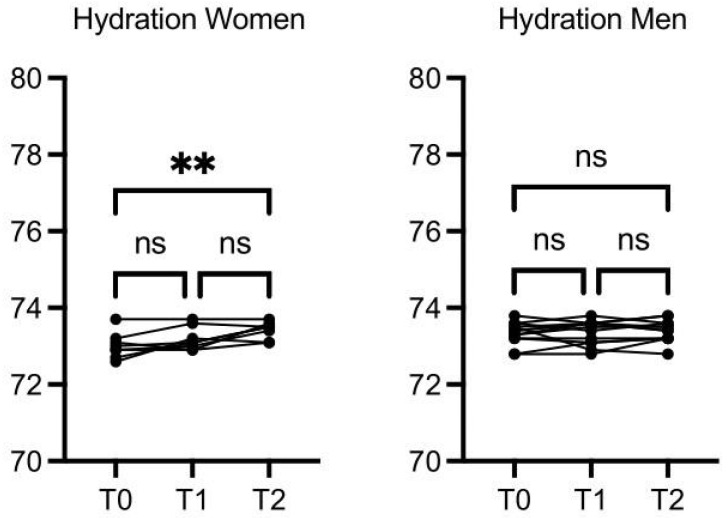
OMWW-OL consumption effects on hydration in volunteers. The images show the single values at T0, T1, and T2. In the women, hydration is increased at T2 vs. T0, while no significant differences were observed in the men. The *Y*-axis reports the values of the analyzed biomarker. Statistics are performed in comparison between the average of T0/T1 ± SEM, T1/T2 ± SEM T0/T2 ± SEM. ** *p* < 0.01, ns: non-significant.

**Figure 4:**
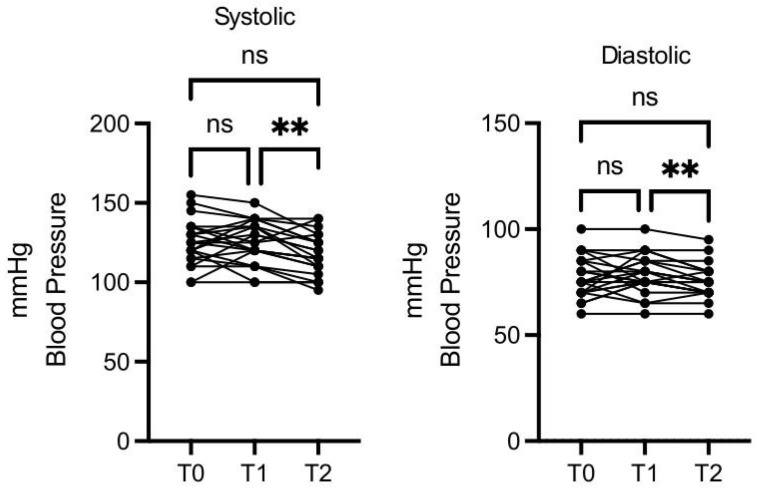
OMWW-OL consumption effects on blood pressure in volunteers. The images show the single values at T0, T1, and T2. Blood pressure values decreased significantly at T2 vs. T1, both in the maximum and minimum values. The *Y*-axis reports the values of the analyzed biomarker. Statistics are performed in comparison between the average of T0/T1 ± SEM, T1/T2 ± SEM, T0/T2 ± SEM. ** *p* < 0.01, ns: non-significant.

**Figure 5:**
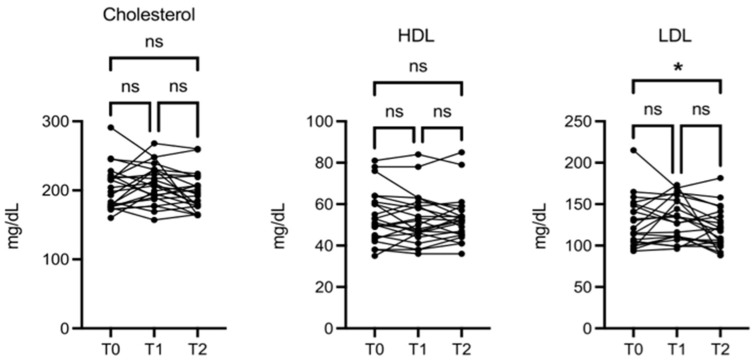
OMWW-OL consumption effects on cholesterol in volunteers. The images show the single values at T0, T1, and T2. There were no significant differences in total cholesterol and HDL, while the LDL decreased slightly but statistically significantly. The *Y*-axis reports the values of the analyzed biomarker. Statistics are performed in comparison between the average of T0/T1 ± SEM, T1/T2 ± SEM T0/T2 ± SEM. * *p* < 0.05, ns: non-significant.

**Figure 6:**
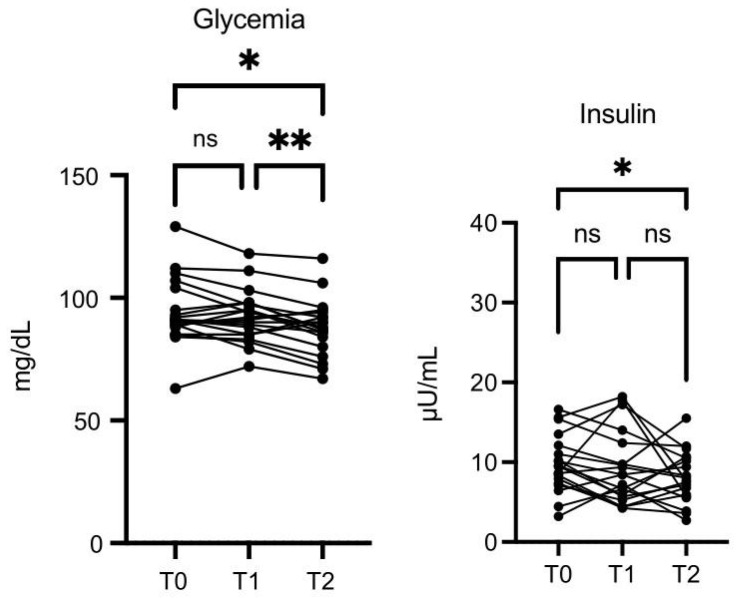
OMWW-OL assumption effects on glycemia and insulin in volunteers. The images show the single values at T0, T1, and T2. Glycemia values decreased at T2 vs. T0, and at T2 vs. T1. Insulin was also decreased at T2 vs. T0. The *Y*-axis reports the values of the analyzed biomarker. Statistics are performed in comparison between the average of T0/T1 ± SEM, T1/T2 ± SEM T0/T2 ± SEM. * *p* < 0.05, ** *p* < 0.01, ns: non-significant.

**Figure 7:**
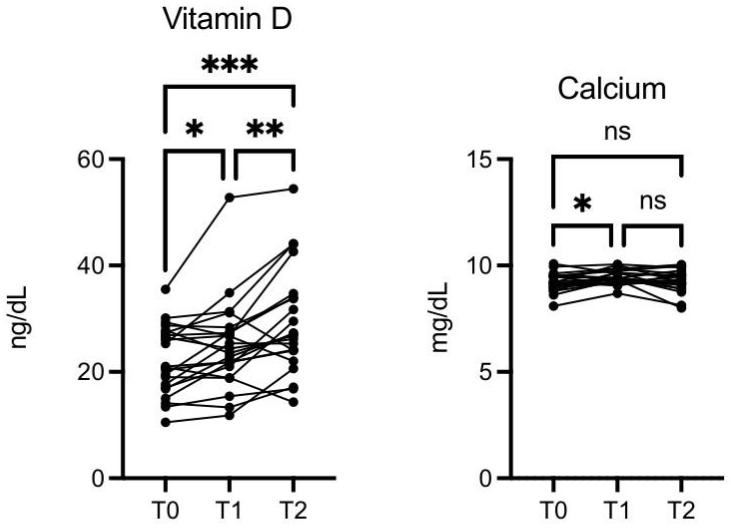
OMWW-OL consumption effects on vitamin D and calcium in volunteers. The images show the single values at T0, T1, and T2. Vitamin D increases following OMWW intake in a statistically significant manner both at T1 and T2. Calcium was increased at T1 vs. T0. The *Y*-axis reports the values of the analyzed biomarker. Statistics are performed in comparison between the average of T0/T1 ± SEM, T1/T2 ± SEM T0/T2 ± SEM. * *p* < 0.05, ** *p* < 0.01, *** *p* < 0.001, ns: non-significant.

**Figure 8:**
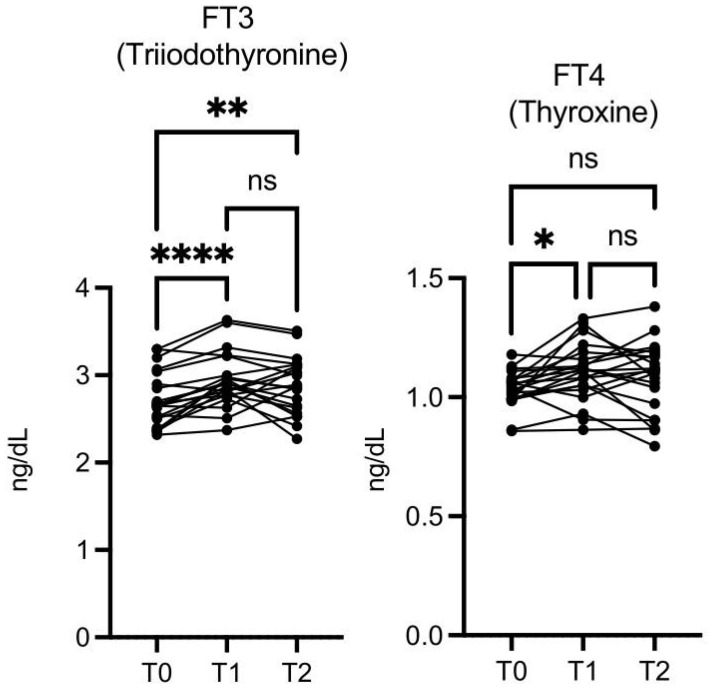
OMWW-OL consumption effects on free thyroid hormones (FT3 and FT4) in volunteers. The images show the single values at T0, T1, and T2. The FT3 level was increased significantly at T1 vs. T0 and at T2 vs. T0, while FT4 was significantly higher at T1 vs. T0. The *Y*-axis reports the values of the analyzed biomarker. Statistics are performed in comparison between the average of T0/T1 ± SEM, T1/T2 ± SEM T0/T2 ± SEM. * *p* < 0.05, ** *p* < 0.01, **** *p* < 0.0001, ns: non-significant.

**Figure 9:**
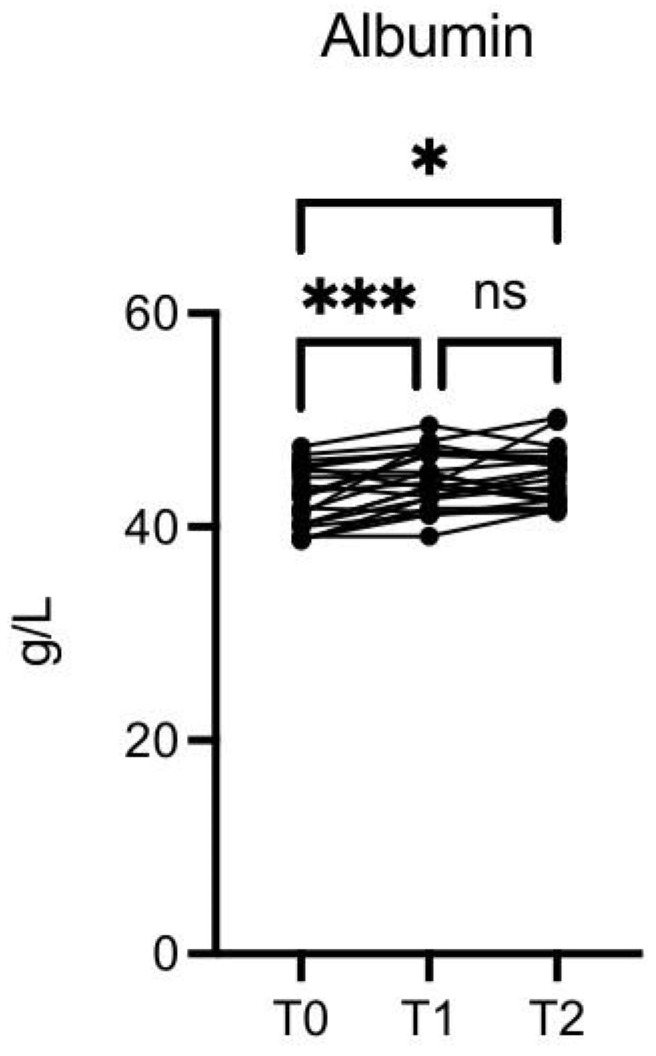
OMWW-OL consumption effects on albumin in volunteers. The images show the single values at T0, T1, and T2. The albumin values increase significantly at T1 vs. T0 and T2 vs. T0. The *Y*-axis reports the values of the analyzed biomarker. Statistics are performed in comparison between the average of T0/T1 ± SEM, T1/T2 ± SEM T0/T2 ± SEM. * *p* < 0.05, *** *p* < 0.001, ns: non-significant.

A total of 29 participants were enrolled in this study; 17 were men, and the mean age was 57 years (ranging from 30 to 72 years). Six participants dropped out of the study after the 30-day time point for personal reasons, and 23 completed the study (average age: 59 years). For better visualization, the parameters that showed significant differences, as well as those with an interesting trend, upon the intervention are reported in graphics (Figure 2, Figure 3, Figure 4, Figure 5, Figure 6, Figure 7, Figure 8 and Figure 9; Figures S1 and S2).

The weight (kg) and BMI did not increase and in female subjects actually decreased slightly but significantly at 30 days (T1) and went back to the original value at 60 days (T2), while no significant changes were observed in the men’s weight and BMI (Figure 2).

The women’s hydration was increased at T2 vs. T0 (*p* ≤ 0.01), while hydration in the men was increased slightly but not in a statistically significant manner (Figure 3).

Blood pressure decreased in a statistically significant manner, both for minimum and maximum values, at T2 vs. T1, while the T1 vs. T0 and T2 vs. T0 differences were not significant (Figure 4).

There was also a change, although not significant, in HDL, and a slight but significant decrease in LDL (Figure 5).

Glycemia values were lower at T2 vs. T0 (*p* ≤ 0.05), and the decrease was more significant at T2 vs. T1 (*p* ≤ 0.01) (Figure 6). Insulin was also decreased at T2 vs. T0 (*p* = 0.05) (Figure 6).

The vitamin D level was significantly increased at T1 vs. T0 (*p* ≤ 0.05), T2 vs. T0 (*p* ≤ 0.001), and T2 vs. T1 (*p* ≤ 0.01) (Figure 7). Calcium was also increased at T1 vs. T0 (*p* ≤ 0.05, Figure 7).

The free triiodothyronine (FT3) level was significantly higher at T1 vs. T0 and at T2 vs. T0, while free thyroxine (FT4) was significantly higher only at T1 vs. T0 (Figure 8). 

The albumin level was significantly increased at T1 vs. T0 (*p* ≤ 0.001) and T2 vs. T0 (*p* ≤ 0.05) (Figure 9).

In our investigation, transferrin, ferritin, and sideremia (Figure S1), and magnesium (Figure S2) did not show variations. Potassium significantly decreased at T2 vs. T1 (Figure S2).

### 3.2. Mass Spectrometry Serum Characterization

**Figure 10:**
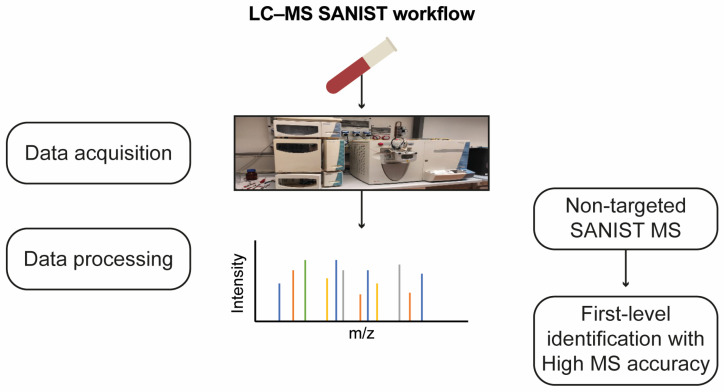
Workflow for non-targeted LC-MS SANIST metabolites profiling.

SANIST profiling revealed three chromatographic peak–area ratios, correlating them with enzyme functionality alteration. The detected potential biomarkers were identified using the approach shown in Figure 10.

**Figure 11:**
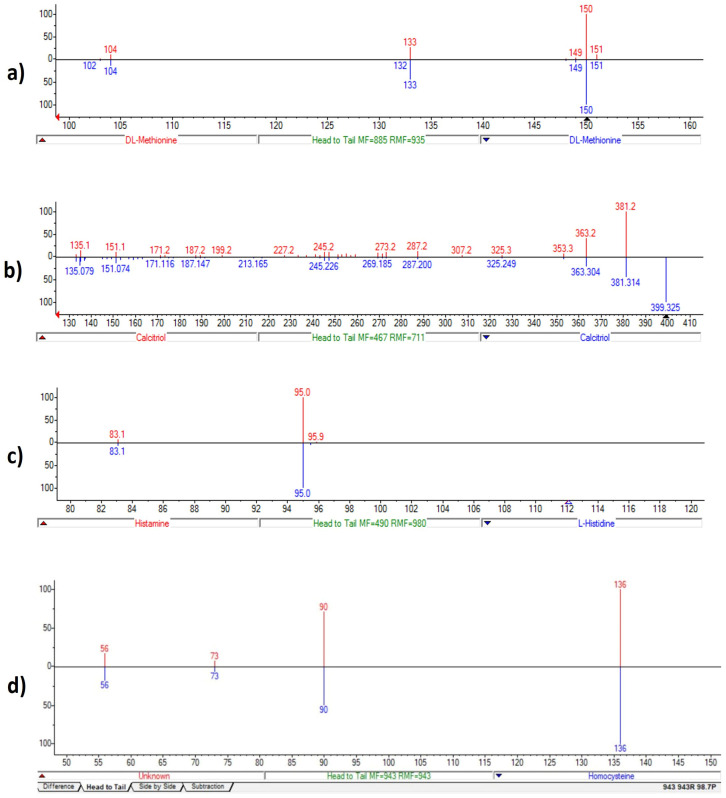
Biomarkers detected by NIST MS Search Program. Tandem mass spectra (MS/MS) library similarity match obtained by LC-MS data using NIST database: D-L-Methionine profile (**a**); Calcitriol (1α,25-dihydroxyvitamin D3) profile (**b**); Histamine profile (**c**), and Homocysteine profile (**d**).

Mass spectra comparisons for the LC-MS data obtained with the NIST library are shown in Figure 11. SANIST technology [26] was used to obtain and compare metabolomics profiles. An Ultimate 3000 UPLC (ThermoFisher) LC system fitted with an Orbitrap mass spectrometer (ThermoFisher) coupled to a surface-activated chemical ionization (SACI)/ESI source was used to analyze the metabolic profiling in both ESI-positive and ESI-negative ion modes.

The ion at *m*/*z* 150 → 133 (loss of a –OH group) (red) was identified by NIST as methionine (C_5_H_11_NO_2_S), present in the library spectrum (blue), with a 92% identification score (direct match factor: 885; reverse match factor: 935) (Figure 11a). Methionine was confirmed by comparison to LC and MS/MS data acquired for the certified analytical standard, following the EU directive (EU directive 2002/657/EC). The ions *m*/*z* 4.0 → 381 and 112 → 95 were identified using the same approach and corresponding to calcitriol and histamine, respectively (Figure 11b,c). We also identified homocysteine with ion at *m*/*z* 136 → 90 (Figure 11d).

Furthermore, we observed a peak at *m*/*z* 371 but the corresponding metabolite was not identified using both an MS and an MS/MS database search. However, this unknown metabolite is very interesting because, according to the similarity approach in MS/MS search, it appears to be highly similar to a peptide molecule. Due to the absence of a commercial internal standard, verifying the data using EU Directive 2002/657/EC was not possible. In order to determine whether the metabolite at *m*/*z* 371 was present in the dietary supplement, OMWW-OL was analyzed using the LC-SANIST-MS/MS method. Nonetheless, it was evidently absent from the analysis of 10 OMWW-OL samples.

We observed a 10-fold increase in the homocysteine/methionine ratio (*m*/*z* 134/150) (*p* = 4 × 10^−5^), the vitamin 25 (OH) D3/histamine ratio (*m*/*z* 401/112) (*p* = 5 × 10^−5^), and the vitamin 25 (OH) D3/not identified metabolite ratio (*m*/*z* 401/371) (*p* = 6 × 10^−5^) with respect to T0.

The mass spectrometry data show very promising beneficial effects on metabolites, with the discovery of a new molecule induced during treatment.

## 4. Discussion

EVOO is an essential Mediterranean diet component rich in bioactive compounds [30]. Several studies suggest the role of its consumption in reducing the incidence of different diseases, such as cancer, cardiovascular pathologies, type 2 diabetes, and neurodegenerative disorders [31,32,33,34,35]. Every year, olive tree cultivation and oil extraction generate a large amount of byproducts, primarily OMWW, which can have a negative environmental impact. However, OMWW is rich in phenolic compounds displaying potent antioxidant, anti-inflammatory, vascular protective, and anti-aging properties [36,37,38,39], and it could potentially be useful in the pharmaceutical and food industries [30,38,40,41,42,43,44,45].

Here, we analyzed the effects of OMWW in a dietary supplement preparation (Oliphenolia^®^, OMWW-OL) on a cohort of volunteers with characteristics close to metabolic syndrome. Our data showed that OMWW-OL intake for 30 days had no negative effects on liver and kidney function and did not affect magnesium levels and iron absorption, transport, and storage. Several studies observed that diets, including daily EVOO, are effective for weight loss [46,47,48]; however, data on women were still lacking. Interestingly, our work found that OMWW-OL was associated with a small decrease in the female participants’ weight and BMI, even in a short time. Furthermore, the finding that hydration was significantly increased in females is a very promising result, as dehydration can significantly impair cognitive and physical abilities and has been associated with obesity, chronic diseases, and decreased longevity [49,50]. OMWW-OL might have the potential to ameliorate the state of hydration. Interestingly, hydration, weight, and BMI are more regulated in women than men, and in accordance with recent literature, including from our group, gender differences in aging parameters were revealed [51]. Hence, although supplementation with OMWW-OL was assessed in a pilot study and for a short time, the disparity between men and women could have biological bases.

In agreement with previous observations, we found that OMWW-OL reduces blood pressure. This effect is probably related to its high polyphenol content [52,53], which would also confer potential cardioprotective properties, as observed for EVOO [54]. We also detected a glycemia decrease after OMWW-OL supplementation in our small cohort. This is consistent with the results obtained by Violi et al. [55], who showed that the supplementation of EVOO at lunch is linked to lower post-prandial glycemic levels. Albeit at a low significance level, cholesterol LDL showed a decreasing trend, while glycemia and insulin decreased significantly. Besides its role in diabetes, insulin increases cancer risk by promoting the proliferation of normal, preneoplastic, and neoplastic cells [56]. By decreasing glycemia and insulin levels, OMWW-OL could have some potential beneficial effects in preventing metabolic syndrome and cancer. More significant differences might be detectable upon supplementation for longer periods and in a larger cohort.

After nutritional intervention with OMWW-OL, we observed an increase in the FT3 and FT4 serum levels, while TSH levels remained stable. Since low FT3 levels are associated with aging [57], it is possible to speculate about the ability of OMWW-OL to increase FT3 and FT4 and to subsequently exert an anti-aging effect, as shown in a previous publication of one of our institutions [58]. In this study, we also observed a significant increase in albumin values. Albumin is involved in many bioactive functions, such as extracellular antioxidant defenses [59] and the transport of various ligands [60,61]. Decreases in albumin levels are associated with an inflammatory state [62] and are observed in cancer, infections, malnutrition, liver illness, and kidney disease [63]. Conversely, high albumin concentrations may protect against early glycemic deterioration and type 2 diabetes progression [64]. Therefore, the increased albumin levels observed with OMWW-OL could correlate with the anti-inflammatory activity of this olive byproduct and contribute to some extent to metabolic syndrome prevention.

Since we observed a potassium reduction, this might be an interesting result for patients with chronic kidney disease, who are typically recommended to reduce potassium consumption or eliminate it from their blood [65,66,67], also considering the absence of any relevant kidney toxicity in the short time of supplementation. Nevertheless, the effect on kidney function should be evaluated upon supplementing OMWW-OL for longer timeframes. Several works have observed that vitamin D appears to be an effective agent to prevent the production of reactive oxygen species and stop cytokine-mediated inflammation [68,69,70,71]. However, the literature does not provide much information on the impact of natural compounds on endogenous vitamin D levels. Our finding that a significant increase in vitamin D, which was not due to seasonal variation, is achieved by supplementing with OMWW-OL supports a previous observation that in vitro digestion using olive oil resulted in higher absorption of the vitamin D precursor, calcifediol, than using other types of edible oils [72]. Since this nutritional intervention also led to increased calcium levels, based on our results it is possible to speculate that OMWW-OL may regulate the calcium balance either by inducing calcium absorption directly or by increasing vitamin D. Calcium is essential for preserving physiological functions and preventing aging, by promoting bone mineralization and sustaining heartbeat, blood clotting, and muscle and neuron function [73,74,75,76]. Naturally, calcium levels decline as we age, and calcium deficiency has been increasingly linked to aging [74,75,77].

Further insights into the dietary intervention were achieved through MS, and differences were observed in the methionine cycle, which involves the regeneration of methionine from homocysteine [78]. The low homocysteine/methionine ratio found in our metabolomic analysis could be associated with active homocysteine metabolism, which is involved in the effective prevention of both oxidative stress and infectious diseases [79]. Elevated homocysteine levels increase the frequency of cardiovascular disease [80,81,82,83,84] with which metabolic syndrome is associated [85]. Therefore, also according to the decreased cardiovascular risk previously observed for olive oil [86], OMWW-OL could reduce cardiovascular risk by lowering homocysteine levels.

The absence of the metabolite at *m*/*z* 371.192 in OMWW-OL suggests that the detection of this metabolite in vivo is due to reduced vitamin D25 oxidation. Therefore, the potential antioxidant activity of OMWW-OL may preserve vitamin D25 and its functionality. Interestingly, the vitamin D/histamine ratio is also high, suggesting a decrease in histamine, an inflammatory mediator [87,88,89]. Since vitamin D regulates the immune system, particularly mast cells that carry histamine [90,91], the decrease in histamine observed with OMWW-OL intervention could be a secondary effect of the vitamin D increase.

In general, the data show that the vital parameters remain in the standard range, showing that, at least in the short time, there is no detrimental effect, with some positive encouraging trends to health benefits.

## 5. Conclusions

Here, we have shown that OMWW-OL has no detrimental effect on a variety of parameters, and also that in a preliminary way, the study indicates the potential to improve biological parameters in volunteers at risk for metabolic syndrome, according to a trend towards a favorable modulation of several biological parameters. The absence of significant variations in most hematochemical parameters indicates that regular Oliphenolia^®^ ingestion has neither adverse effects on lipid levels nor on liver or kidney functions.

Some aging-associated parameters were modulated favorably. In addition to extending the period without needing treatment and slowing the aging process, it could be hypothesized that OMWW-OL could help prevent or postpone the onset of age-related disorders, improving quality of life and reducing healthcare costs. MS data revealed improved vitamin D/histamine, homocysteine/methionine, and vitamin D/unknown metabolite ratios.

Our data suggest that OMWW-OL may also have a promising effect in protecting cells from reactive oxygen species-induced injuries due to its high polyphenol content and vitamin D-boosting effect. The possibility of targeting aging instead of treating age-related pathologies is very attractive, as it can improve the quality of life and ensure longevity. This approach would align with positive biology, which researches the biological mechanisms involved in well-being [92]. Some of these parameters could also be relevant in cancer prevention or interception.

This study has some limitations, such as the small sample size, the single-arm protocol (thus lacking an external control group), and the short duration of the follow-up. Therefore, the information must be considered preliminary, although encouraging, and needs to be strengthened in further research with a longer supplementation time and follow-up period and an independent control group.

Future studies are needed to better elucidate the structure of the potential new metabolite and to investigate biological correlates.

## Data Availability

The data that support the findings of this study are available on request from the corresponding author.

## Supplementary Materials

The following are available online at https://www.mdpi.com/article/10.3390/nu16183068/s1, Figure S1: Effects of OMWW-OL consumption on iron parameters in volunteers, Figure S2: Effects of OMWW-OL consumption on potassium and magnesium values in volunteers.
