# Supplementary material for: Effect of a Phytochemical-Rich Olive-Derived Extract on Anthropometric, Hematological, and Metabolic Parameters
Source: Nutrients. 2024 Sep 11;16(18):3068. doi: 10.3390/nu16183068 (PMC11435251; doi:10.3390/nu16183068)
Supplement: Supplementary file 1 [file nutrients-16-03068-s001.zip › nutrients-3161072-supplementary.pdf]

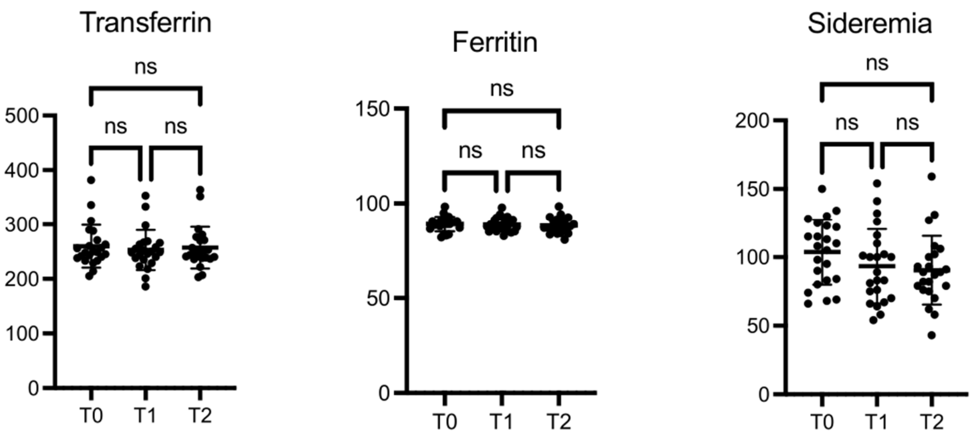

Figure S1. Effects of OMWW-OL consumption on iron parameters in volunteers.

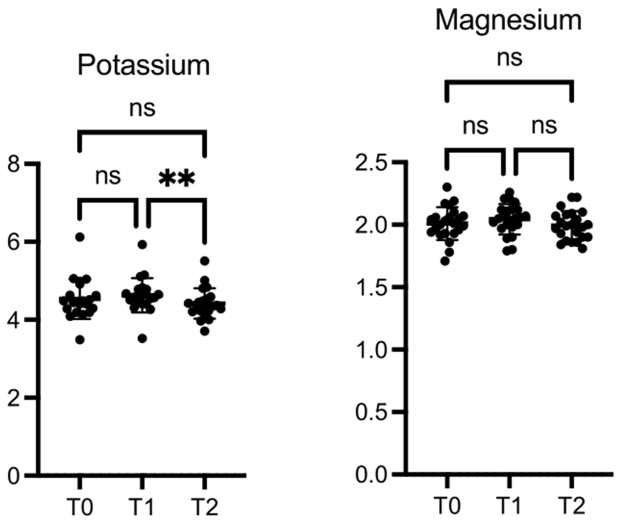

Figure S2. Effects of OMWW-OL consumption on potassium and magnesium values in volunteers.
